# Effects of Congenital Adrenal Hyperplasia (CAH) and Biological Sex on Brain Size

**DOI:** 10.3390/anatomia3030012

**Published:** 2024-07-18

**Authors:** Eileen Luders, Christian Gaser, Debra Spencer, Ajay Thankamony, Ieuan Hughes, Umasuthan Srirangalingam, Helena Gleeson, Melissa Hines, Florian Kurth

**Affiliations:** 1 Department of Women’s and Children’s Health, Uppsala University, 75237 Uppsala, Sweden; 2 Swedish Collegium for Advanced Study (SCAS), 75238 Uppsala, Sweden; 3 School of Psychology, University of Auckland, Auckland 1010, New Zealand; 4 Laboratory of Neuro Imaging, School of Medicine, University of Southern California, Los Angeles, CA 90033, USA; 5 Department of Neurology, Jena University Hospital, 07747 Jena, Germany; 6 Department of Psychiatry and Psychotherapy, Jena University Hospital, 07747 Jena, Germany; 7 Department of Psychology, University of Cambridge, Cambridge CB2 3RQ, UK; 8 Department of Paediatrics, Addenbrooke’s Hospital, University of Cambridge, Cambridge CB2 0QQ, UK; 9 Weston Centre for Paediatric Endocrinology & Diabetes, Addenbrooke’s Hospital, University of Cambridge, Cambridge CB2 0QQ, UK; 10 Department of Endocrinology and Diabetes, University College Hospital London, London NW1 2BU, UK; 11 Queen Elizabeth Hospital, Birmingham B15 2WB, UK; 12 Department of Diagnostic and Interventional Radiology, Jena University Hospital, 07747 Jena, Germany

**Keywords:** androgens, CAH, MRI, ICV, sex, TBV

## Abstract

Congenital Adrenal Hyperplasia (CAH) has been reported to involve structural alterations in some brain regions. However, it remains to be established whether there is also an impact on the size of the brain as a whole. Here, we compiled the largest CAH sample to date (n = 53), matched pair-wise to a control group (n = 53) on sex, age, and verbal intelligence. Using T1-weighted brain scans, we calculated intracranial volume (ICV) as well as total brain volume (TBV), which are both common estimates for brain size. The statistical analysis was performed using a general linear model assessing the effects of CAH (CAH vs. controls), sex (women vs. men), and any CAH-by-sex interaction. The outcomes were comparable for ICV and TBV, i.e., there was no significant main effect of CAH and no significant CAH-by-sex interaction. However, there was a significant main effect of sex, with larger ICVs and TBVs in men than in women. Our findings contribute to an understudied field of research exploring brain anatomy in CAH. In contrast to some existing studies suggesting a smaller brain size in CAH, we did not observe such an effect. In other words, ICV and TBV in women and men with CAH did not differ significantly from those in controls. Notwithstanding, we observed the well-known sex difference in brain size (12.69% for ICV and 12.50% for TBV), with larger volumes in men than in women, which is in agreement with the existing literature.

## 1. Introduction

Congenital Adrenal Hyperplasia (CAH) is a genetic disorder that affects the adrenal glands and involves alterations in glucocorticoids and androgens [1]. CAH has also been reported to be associated with structural changes in some brain regions [2,3]. However, it is not clear yet whether brain size per se is different in individuals with CAH.

Out of eleven CAH studies based on structural neuroimaging [2], at least four assessed brain size [1,4-6], measured as intracranial volume (ICV), total brain volume (TBV), or “total cerebral volume” (which resembles TBV). Two of these studies [4,5] seem to suggest that CAH is accompanied by a smaller brain size: The first study [4] examined TBV and included 27 children and adolescents with CAH (16 females/11 males) and 35 healthy controls (20 females/15 males), aged 8–18 years. The second study [5] examined ICV and included a larger and slightly older cohort, aged 16–33 years, consisting of 37 individuals with CAH (21 females/16 males) and 43 healthy controls (26 females/17 males). In contrast, the two other studies [1,6] measuring TBV (or “total cerebral volume”) in 19 women with CAH and 19 control women, aged 18–50 years [6], and in 27 children with CAH and 47 control children, aged 6–16 years [1], reported a lack of significant differences in brain size in CAH. Interestingly though, the latter study [1] observed a trend toward decreased cerebral volumes in girls with CAH compared to control girls but not in boys with CAH compared to control boys.

To further advance an understudied field of research we examined ICV as well as TBV in a large cohort of individuals with CAH. In addition to testing for a significant main effect of CAH and a significant CAH-by-sex interaction, we tested for a significant main effect of sex because prior research indicated smaller brain volumes in females than males, independent of CAH [7-10].

## 2. Materials and Methods

### 2.1. Study Sample

The sample consisted of 53 individuals (33 women and 20 men) with classic CAH [11], aged between 18 and 46 years (mean ± SD: 30.15 ± 7.92 years), and 53 controls (33 women and 20 men), aged between 18 and 45 years (mean ± SD: 30.34 ± 7.71 years). Of the 53 individuals with CAH, 29 presented with a salt-wasting phenotype and 18 with a simple virilizing phenotype; the remaining 6 individuals with CAH did not have information on the form of the condition. Individuals with CAH were pair-wise matched to controls with respect to sex, age, and education, as well as verbal skills (as a proxy for general intelligence), as determined using the Advanced Vocabulary Test [12]. All participants were required to be free from neurological or psychiatric disorders and to have no contraindications to magnetic resonance imaging (MRI). The study was approved by a National Health Service Research Ethics Committee and the Health Research Authority in the United Kingdom (15/EM/0532) as well as the Ethics Committee at the University of Auckland in New Zealand (020825). All participants provided their informed consent.

### 2.2. Image Acquisition and Processing

Structural T1-weighted images of the brain were acquired from each participant on a Siemens 3.0 Tesla Skyra system with a 32-channel head coil using the following parameters: TR = 2300 ms, TE = 2.98 ms, flip angle = 9°, matrix size = 256 × 240, 176 sagittal sections, and voxel size = 1 × 1 × 1 mm^3^. All brain images were processed via the CAT12 toolbox [13], version 12.6, and SPM12, version r7771, as detailed elsewhere [13-16]. More specifically, images were first denoised by a spatially adaptive non-local means filter [17], corrected for magnetic field inhomogeneities, and then skull-stripped [18]. This was followed by an adaptive maximum a posteriori tissue segmentation [19], which also included a partial volume estimation [20]. Finally, the resulting tissue segments, including gray matter (GM), white matter (WM), and cerebrospinal fluid (CSF), were used to calculate both ICV (GM + WM + CSF) and TBV (GM + WM).

### 2.3. Statistical Analysis

The statistical analysis was performed using a general linear model to assess the effects of CAH (CAH vs. controls), sex (female vs. male), and any CAH-by-sex interaction. ICV and TBV constituted the dependent variables, whereas CAH status, sex, and the CAH-by-sex interaction were the independent variables. Significance was established at *p* ≤ 0.05 using Monte Carlo simulations with 10,000 permutations to avoid relying on assumptions for parametric testing. In addition, we conducted supplementary analyses, separately for ICV and TBV, testing for a significant main effect of CAH or any CAH-by-sex interactions when splitting CAH by phenotype: salt-wasting form vs. simple virilizing form.

## 3. Results

### 3.1. Intracranial Volume (ICV)

**Figure 1.**
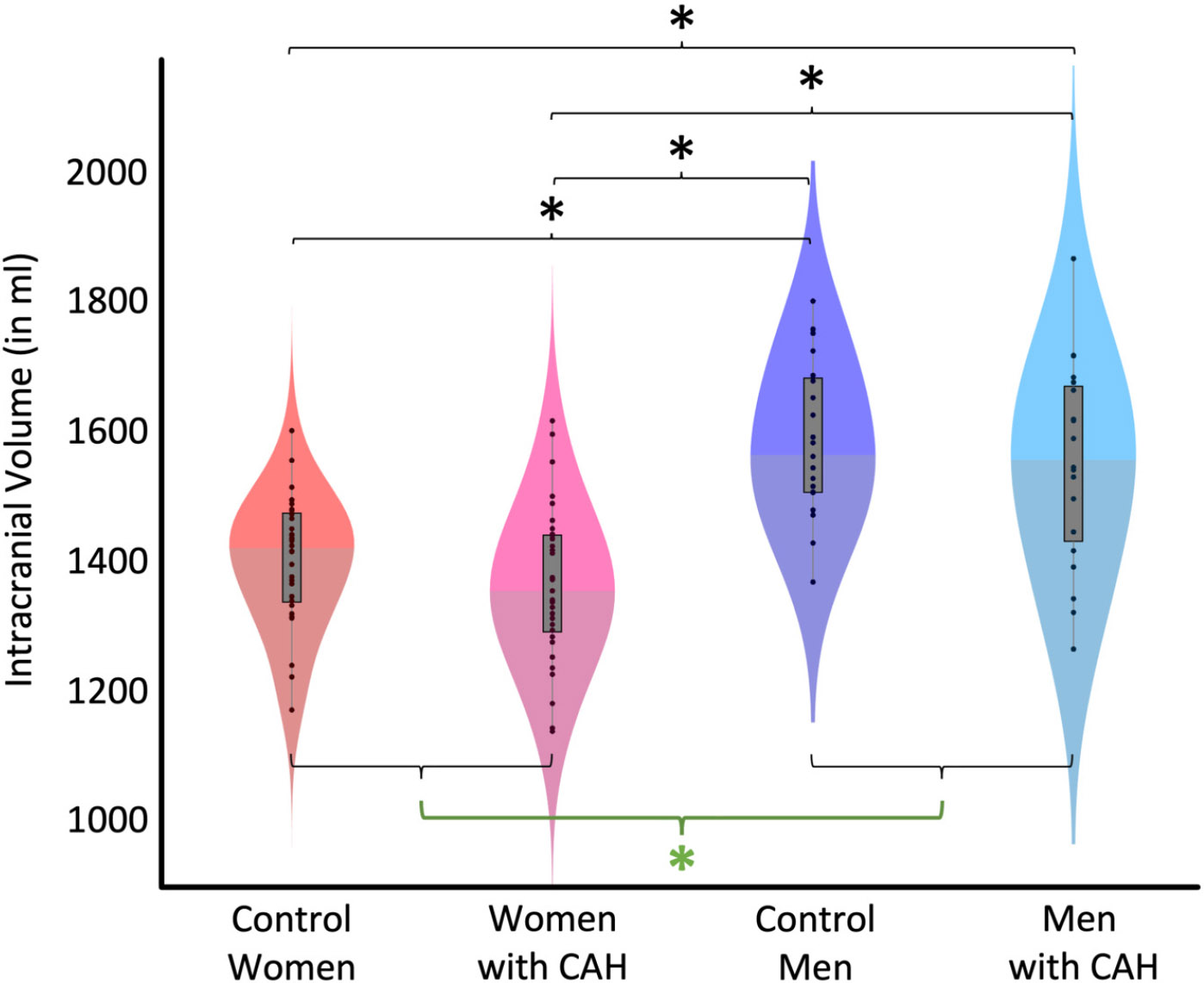
Group-specific intracranial volume (ICV). The violin plots depict ICV for each of the four groups. The black dots show individual volume estimates, the gray boxes show the group-specific interquartile ranges, the whiskers show the group-specific 1.5 interquartile ranges, and the difference in shading indicates the median. The main effect of sex (green asterisk) was significant, with larger ICVs in all males (control men + men with CAH) compared to all females (control women + women with CAH). In addition, male and female subgroups differ significantly from each other (black asterisks).

There was no significant CAH-by-sex interaction (*p* = 0.983; F(1,102) < 0.01) and also no significant main effect of CAH (*p* = 0.127; F(1,102) = 2.35). In contrast, there was a significant main effect of sex (*p* < 0.001; F(1,102) = 60.17), with larger ICVs in men compared to women. The magnitude of the sex difference was 12.69%. Table 1 provides group-specific means and standard deviations. Figure 1 illustrates the group-specific volumes and the significant group differences (main effect of sex as well as post hoc effects).

Given the lack of a significant CAH-by-sex interaction, post hoc tests were not required. Notwithstanding, their results are provided in Table 2 (for ICV) and in Table 4 (for TBV) to provide a reference against which findings can be compared in future studies.

### 3.2. Total Brain Volume (TBV)

**Figure 2.**
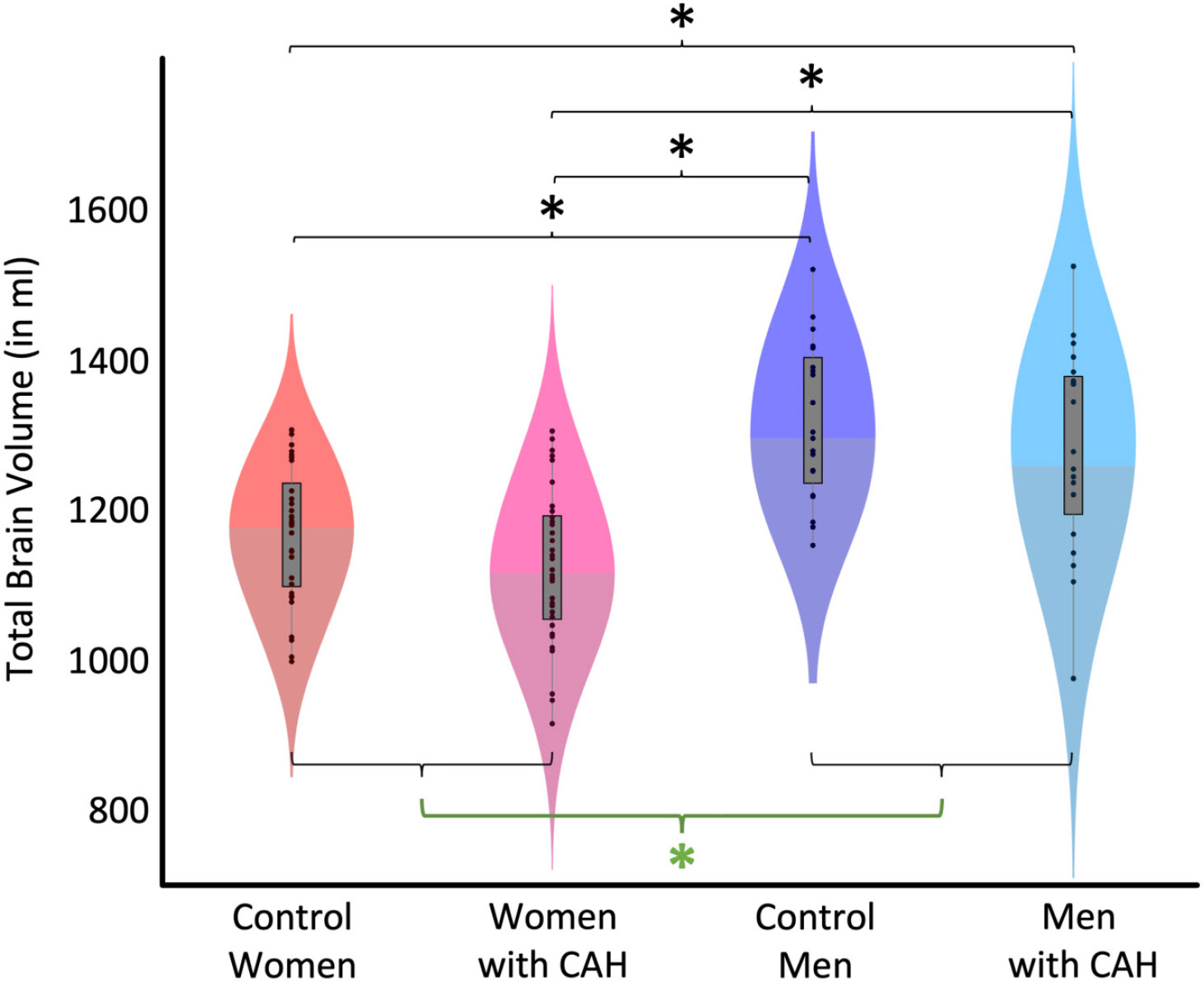
Group-specific total brain volume (TBV). The violin plots depict TBV for each of the four groups. The black dots show individual volume estimates, the gray boxes show the group-specific interquartile ranges, the whiskers show the group-specific 1.5 interquartile ranges, and the difference in shading indicates the median. The main effect of sex (green asterisk) was significant, with larger TBVs in all males (control men + men with CAH) compared to all females (control women + women with CAH). In addition, male and female subgroups differ significantly from each other (black asterisks).

There was no significant CAH-by-sex interaction (*p* = 0.877 F(1,102) = 0.02) and also no significant main effect of CAH (*p* = 0.058; F(1,102) = 3.66). In contrast, there was a significant main effect of sex (*p* < 0.001; F(1,102) = 52.17), with larger TBVs in men compared to women. The magnitude of the sex difference was 12.5%. Table 3 provides group-specific means and standard deviations. Figure 2 illustrates the group-specific volumes and the significant group differences (main effect of sex as well as post hoc effects). Table 4 provides the statistics for the post hoc tests.

### 3.3. Supplementary Analyses (Effect of CAH Phenotype)

In accordance with the results reported above, there was no significant main effect of CAH (neither for ICV nor for TBV) when taking into account the CAH phenotype. In other words, there were no significant differences between individuals with the salt-wasting form and controls, between individuals with the simple virilizing form and controls, or between individuals with the salt-wasting form and individuals with the simple virilizing form. There was also no significant CAH-by-sex interaction.

## 4. Discussion

Our findings contribute to an understudied field of research exploring brain size—by means of ICV and TBV—in 53 individuals with CAH and 53 matched controls, the largest CAH sample to date. We did not detect a significant main effect of CAH or a CAH-by-sex interaction. However, we observed a significant main effect of sex.

### 4.1. No Significant CAH Effect

We did not detect a significant main effect of CAH. In other words, there were no differences in brain size between individuals with CAH and controls, which is in agreement with the outcomes of two other studies [1,6]. A significant main effect of CAH would suggest influences of CAH (e.g., endogenous decreases in glucocorticoids) and/or treatment of CAH (e.g., exogenous increases in glucocorticoids). Interestingly, there are two previous studies that reported a smaller ICV [4] or TBV [5] in CAH. However, the mean age in those latter two studies was considerably lower (12.8 years and 21.7 years, respectively) than in the present study (30.2 years). Thus, it is possible that any brain size deviations in CAH in earlier stages of life normalize later. However, more research is needed to confirm (or deny) if smaller brain sizes are typical for CAH in earlier stages of life at all. For example, in one of the aforementioned studies where brain size was not significantly reduced in children with CAH compared to control children [1], the mean age was even lower (9.8 years). Longitudinal developmental studies would be useful to provide more definitive information but do not exist (yet).

### 4.2. No Significant CAH-by-Sex Interaction

We did not detect a significant CAH-by-sex interaction. The presence of a CAH-by sex interaction (e.g., differences between women with CAH and control women, but not between men with CAH and control men) would suggest influences of increased prenatal androgens in female brains because classic CAH causes elevated androgen levels in females but not males [3]. While prenatal androgens or sex steroids in general, among other factors [21-23], have been proposed to play a significant role in determining (sexually dimorphic) brain features [24], their impact might be more enhanced on the regional level affecting certain brain structures (e.g., the amygdala) with a high density of sex steroid receptors [25], rather than brain size as a whole. Follow-up studies will further enhance this field of research by focusing on the volumes of selected brain regions or by exploring other brain features (e.g., local gray matter or cortical thickness) using morphometric measures that cover the entire brain/cortex with a high regional specificity (e.g., voxel-wise or vertex-wise).

### 4.3. Significant Sex Effect

We detected a significant main effect of sex effect, with larger brain sizes in men compared to women. The magnitude of the sex difference (12.69% for ICV and 12.50% for TBV) is comparable with what has been reported in the normative literature [8,26]. The observed effect suggests influences of genes located on the sex chromosomes, influences of sex steroids, or influences of the environment [22]. As discussed elsewhere [23], genes on the sex chromosomes are likely to contribute to the brain’s sexually dimorphic phenotype in two ways: directly by acting in the brain itself (differentiating XX and XY brain cells) and indirectly by acting on the gonads (regulating gonadal secretions that have sex-specific effects on the brain). Sex differences in global brain and tissue volumes are present already in neonates and infants [27,28]. So, in theory, genes and prenatal sex steroids may have an impact on brain size. However, given that the impact of sex steroids, specifically prenatal androgens, seems to be minute (as there were no differences between women with CAH and control women, see Section 4.2), genes might play the more dominant role in determining brain size, at least early in life [29,30]. Later in life, environmental influences (e.g., the differential effect of sex-specific social environments; see [22]) as well as postnatal sex steroids may exert additional effects. This is supported by studies reporting a widening of the sex difference over time for various brain measures, including brain size [31-37].

## Figures and Tables

**Table 1.** Descriptive statistics for ICV (in mL): mean ± standard deviation.

Control Women	Women with CAH	Control Men	Men with CAH	All Women	All Men
1399.06	1361.53	1585.52	1549.07	1380.29	1567.30
±99.05	±117.84	±118.74	±153.96	±109.65	±136.96

**Table 2.** Post hoc group comparisons for ICV.

	Effect Size (Cohen’s d)	t (df)	Significance (*p*)
Control Women vs. Control Men	−1.08	−5.47 (102)	<0.001
Control Women vs. Women with CAH	0.25	1.27 (102)	0.166
Women with CAH vs. Men with CAH	−1.09	−5.50 (102)	<0.001
Control Men vs. Men with CAH	0.19	0.96 (102)	0.407
Women with CAH vs. Control Men	−1.30	−6.57 (102)	<0.001
Control Women vs. Men with CAH	−0.87	−4.40 (102)	<0.001

**Table 3.** Descriptive statistics for TBV (in mL): mean ± standard deviation.

Control Women	Women with CAH	Control Men	Men with CAH	All Women	All Men
1167.09	1123.43	1316.61	1279.37	1145.26	1297.99
±88.08	±102.30	±104.44	±134.87	±97.24	±120.55

**Table 4.** Post hoc group comparisons for TBV.

	Effect Size (Cohen’s d)	t (df)	Significance (*p*)
Control Women vs. Control Men	−0.99	−5.00 (102)	<0.001
Control Women vs. Women with CAH	0.33	1.68 (102)	0.166
Women with CAH vs. Men with CAH	−1.03	−5.21 (102)	<0.001
Control Men vs. Men with CAH	0.22	1.12 (102)	0.407
Women with CAH vs. Control Men	−1.28	−6.46 (102)	<0.001
Control Women vs. Men with CAH	−0.74	−3.75 (102)	<0.001

## Data Availability

The data are not publicly available due to ethical restrictions imposed by the signed consent.
